# Sexual Dimorphism of the Feto-Placental Phenotype in Response to a High Fat and Control Maternal Diets in a Rabbit Model

**DOI:** 10.1371/journal.pone.0083458

**Published:** 2013-12-26

**Authors:** Anne Tarrade, Delphine Rousseau-Ralliard, Marie-Christine Aubrière, Nathalie Peynot, Michèle Dahirel, Justine Bertrand-Michel, Tiphaine Aguirre-Lavin, Olivier Morel, Nathalie Beaujean, Véronique Duranthon, Pascale Chavatte-Palmer

**Affiliations:** 1 INRA, UMR1198 Biologie du Développement et Reproduction, Jouy-en-Josas, France; 2 ENVA, Maisons Alfort, France; 3 PremUp Foundation, Paris, France; 4 INSERM, UMR 1048- Institut des Maladies Métaboliques et Cardiovasculaires, Toulouse, France; Otto-von-Guericke University Magdeburg, Germany

## Abstract

Maternal environment during early developmental stages plays a seminal role in the establishment of adult phenotype. Using a rabbit model, we previously showed that feeding dams with a diet supplemented with 8% fat and 0.2% cholesterol (HH diet) from the prepubertal period and throughout gestation induced metabolic syndrome in adult offspring. Here, we examined the effects of the HH diet on feto-placental phenotype at 28 days post-coïtum (term = 31days) in relation to earlier effects in the blastocyst (Day 6). At 28 days, both male and female HH fetuses were intrauterine growth retarded and dyslipidemic, with males more affected than females. Lipid droplets accumulated in the HH placentas’ trophoblast, consistent with the increased concentrations in cholesteryl esters (3.2-fold), triacylglycerol (2.5-fold) and stored FA (2.12-fold). Stored FA concentrations were significantly higher in female compared to male HH placentas (2.18-fold, p<0.01), whereas triacylglycerol was increased only in HH males. Trophoblastic lipid droplet accumulation was also observed at the blastocyst stage. The expression of numerous genes involved in lipid pathways differed significantly according to diet both in term placenta and at the blastocyst stage. Among them, the expression of *LXR*-*α* in HH placentas was reduced in HH males but not females. These data demonstrate that maternal HH diet affects the blastocyst and induces sex-dependent metabolic adaptations in the placenta, which appears to protect female fetuses from developing severe dyslipidemia.

## Introduction

Non-communicable diseases such as obesity, diabetes, dyslipidemia and hypertension are a main cause of public health concern. Chronic diseases depend on adult lifestyle but also on adverse environmental stimuli encountered in early development. Pregnancy is a sensitive window of vulnerability as described by the Barker’s concept known as Developmental Origins of Health and Disease ( http://www.mrc.soton.ac.uk/dohad/ ) [1]. Epidemiological studies have shown a correlation between low birth weight and cardiovascular disease or type-2 diabetes at adulthood, related to fetal growth restriction [2]. Moreover, studies on the Dutch famine in 1944-45 demonstrated that the occurrence of non-communicable diseases in the offspring depends on the timing of exposure during the prenatal period [3]. In response to an impaired *in utero* environment, the fetus develops adaptive responses to improve its survival. When the antenatal and the post-natal environment are not in conformity, the risk of developing non-communicable diseases at adulthood increases [4].

In the past decades, expanding Western lifestyle habits have induced drastic dietary changes in human populations, especially with increased fat intake. In Europe, the fat consumption by women of childbearing age in the UK reached 35.4% [5] and 39.6% in France, whereas current recommendations indicate that they should be between 30 and 35% [6]. Maternal hypercholesterolemia is associated with enhanced fatty streak formation in fetal arteries, which leads to atherosclerosis during childhood [7]. To evaluate the relationship between maternal fat excess and adult offspring phenotype, animal models have been generated. In rodents, fetal exposure to a maternal high fat diet during pregnancy induced phenotypic outcomes such as dyslipidemia, cardiovascular deregulation, impaired liver lipid metabolism and glucose homeostasis in the adult offspring [5]. In rabbits, diet-induced maternal hypercholesterolemia during pregnancy led to atherosclerotic lesions in fetus [8]. In addition, a maternal lipid- and cholesterol-enriched diet (HH diet) led to offspring overweight associated with hypertension [9]. Altogether, these studies suggest that excess maternal fat intake is deleterious for later life of offspring.

During pregnancy, the preimplantation period has been described to be sensitive to the maternal environment. In the rabbit HH model described above, the maternal HH diet led to abnormal gene expression at the embryonic genome activation stage [9]. In rats, maternal protein restriction during the periconceptional period induced a reduction in blastocyst cell number both within the inner cell mass and the trophectoderm [10], together with subsequent sex-dependent excess growth and hypertension in the offspring. In mice, maternal protein restriction during this period affected the function of the yolk sack, which developed enhanced endocytic capacity to increase nutrient retrieval [11], and induced sex-dependent effects on offspring growth, cardiovascular and adipose tissue phenotype [12]. Environmental conditions encountered by the embryo were thus shown to affect both embryonic and some extra-embryonic lineages, but the effects on the trophoblast have not been explored yet.

The placenta is involved in materno-fetal exchanges, metabolism, endocrinology and immune pathways and is an active component for fetal growth. Any disruption of placental development may alter its structure and function, notably nutrient supply from the mother to the fetus, which controls harmonious fetal growth. Studies from the Helsinki Birth Cohort demonstrate that the relationship between placental surface area and offspring hypertension is dependent on the mothers' nutritional state [13]. Inadequate maternal nutritional environment such as high fat intake before and during pregnancy disturbs placental function through impaired gene expression, vascular regulation, perturbed hemodynamic parameters and inflammation [14]. In rabbits, maternal hypercholesterolemia is known to alter placental lipid composition, cholesterol synthesis and the expression of a glucose transporter [15], [16]. Moreover, it was recently shown in rodents that placental gene expression in response to the maternal environment differs between males and females, leading to distinctive transcriptomic signatures [17], [18].

To date, however, there is no data available connecting blastocyst and feto-placental phenotype and taking into consideration sex effects at the end of gestation. In order to study longitudinal effects related to maternal fat intake, the rabbit model offers several main advantages as recently reviewed [19]. Among those, phylogenetic and genetic comparisons of different mammalian genomes indicate that the rabbit is closer to the human compared to rodents [20]. Moreover, its embryo development is well described and some epigenetic events, such as X inactivation, have been shown to be more similar to humans in rabbits compared to mice [21]. The rabbit genome is sequenced (X7) and genomic tools are available [22]. Due to the historical use of rabbits in toxicology, rabbit physiology is also well known. Moreover, LDL (Low density lipoprotein) concentrations increase in rabbits in response to high fat diets, as described in humans and in contrast to rodents. Finally, the rabbit placenta is of hemodichorial type, which is closer to the human placenta than that of mice and rats [23], [24].

Previous studies in our laboratory have shown that a maternal lipid and cholesterol-enriched diet (HH diet) administered to rabbit does from the prepubertal period and throughout gestation induces differences in gene expression in the embryo at the 8–16 cell stage, subsequent fetal growth retardation and components of the metabolic syndrome in adult offspring [9]. The objective of the present study was to explore the effects of maternal high fat nutrition on feto-placental and blastocyst phenotype using this biologically pertinent rabbit model [19], taking into consideration the effect of the sex of the conceptus at D28 of gestation.

## Methods

### Ethical approval

The experiment was performed in accordance with the International Guiding Principles for Biomedical Research involving Animals as promulgated by the Society for the Study of Reproduction and in accordance with the European Convention on Animal experimentation. The local ethical committee (Comethea, n°45 in French National register) approved the experimental design, which was registered under n°11/037.

### Animals

**Figure 1:**
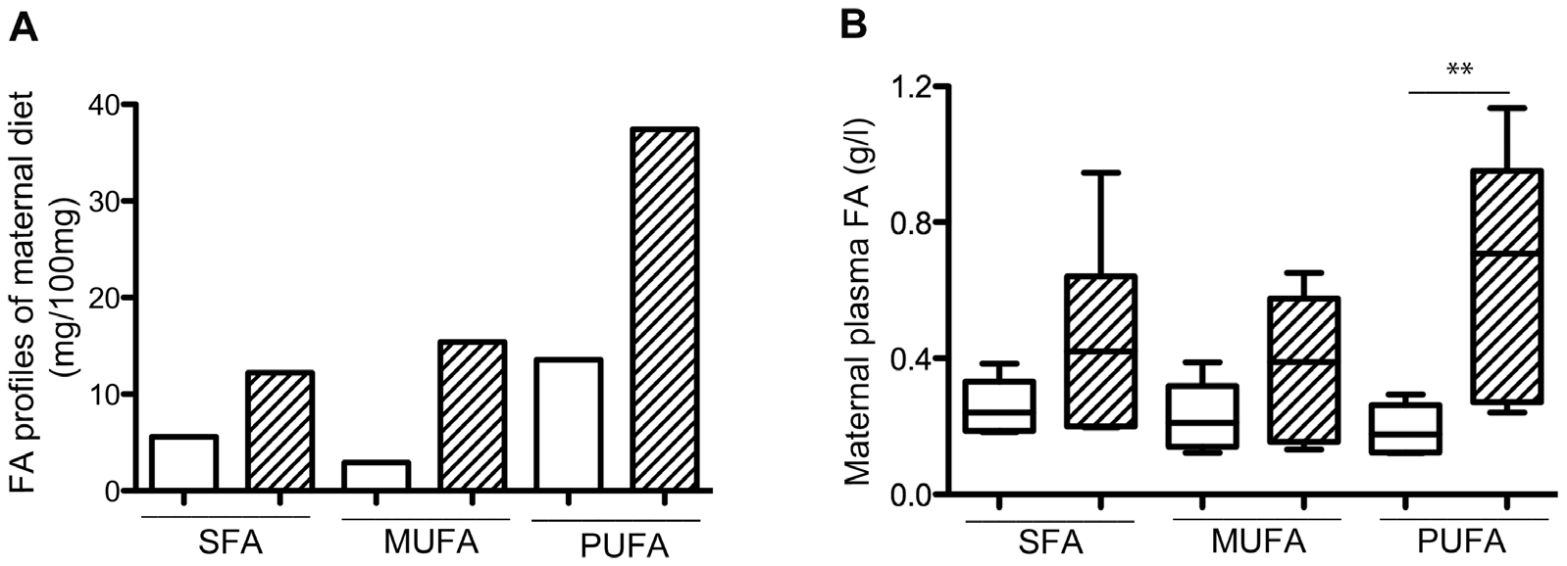
FA profiles of maternal diet (A) and blood (B) according to maternal diet. HH is indicated by hatched columns and C diet by white columns. (A) FA concentrations of maternal diet were expressed in mg/100 g of food. In (B), 6 samples per group were used to evaluate SFA, MUFA and PUFA concentrations. (**p<0.01).

New-Zealand white rabbit does of INRA 1077 line were used. They were housed individually in the same animal holding facilities with temperature and light controlled environment. At 10 weeks of age, does were fed *ad libitum* with either a lipid (8%, including 6% of soybean oil - i.e. predominantly polyunsaturated fatty acids (PUFA)) cholesterol-enriched (0.2%) diet (HH) or a control diet (C, 2% lipids). Nutrient and chemical composition of C and HH diets have been previously published by Picone [9]. Briefly, the control diet (18.08% crude protein, 14.20% crude fiber, 19.43% starch and 2.03% fat) was based on a constant composition of vegetal feedstuff with high fiber contents adapted to rabbit. The experimental HH diet (control diet supplemented with 6% soybean oil and 0.2% cholesterol) contained quantitatively more FA than the C diet from each fatty acid class, mainly n-6 PUFA (Figure 1A) and provided 16% more energy than the C diet.

At 18 weeks of age, to obtain enough blastocysts to perform immunohistochemistry and RT-qPCR, 2HH and 2C does were superovulated by five subcutaneous administrations of pFSH (Stimufol®, Merial) at 12 h intervals as described previously [25], [26], followed 12 h later by an intravenous administration of 30 IU HCG (Chorulon, Intervet) at the time of natural mating. On day 6 of gestation, does were euthanized, blastocysts were recovered from oviducts by flushing, then fixed for immunohistochemistry (n = 41) or dry frozen individually for sex determination and RT-qPCR (n =  24).

At 18 weeks of age, 12 does were mated naturally. On D28 of gestation, i.e. 3 days before the end of the gestation, 7HH and 5C pregnant does were euthanized. Fetuses, placentas, fetal and maternal blood were collected. Fetuses and placentas were weighed. Liver samples were snap-frozen for several biochemical and molecular biological analyses among which the determination of the sex of the fetuses by PCR. Placentas from three does of each group were dissected from the deciduas and the labyrinthine areas were conditioned for transmission electron microscopy, lipid-cholesterol profiles and gene expression. For placenta and liver analyses, one or two male/female couples were selected randomly from three dams of each group to limit a maternal-induced effect.

### Blood samples and clinical biochemistry

Blood from dams and fetuses was collected into EDTA coated vacutainers, centrifuged and supernatant (plasma) was stored at –20°C until analysis. Total cholesterol and triacylglycerol (23 HH and 26 C fetuses) were measured using an enzymatic assay Cholesterol RTU® and TG PAP 150 (BioMerieux, France), respectively.

### Sex determination

For fetuses, DNA was extracted from liver and PCR was performed as described by Diaz-Hernandez [27].

### Structural and ultrastructural histology

Labyrinthine area collected randomly from each of the different litters were fixed with 2% of glutaraldehyde, post-fixed in 1% of osmium tetraoxide and contrasted with uranyl acetate. Samples were dehydrated and embedded in Epon. Sections (0.5 µm) were performed, stained and examined with an Olympus microscope. Ultrathin sections were stained with lead citrate and examined with a Zeiss EM902 EELS transmission electron microscope.

### RNA isolation and RT-qPCR

RNA from each blastocyst (n = 13 HH, n = 11 C) was extracted using the AllPrep DNA/RNA Mini Kit (Qiagen). cDNA synthesis was prepared using 200 ng of RNA and 200U SuperScript III Reverse transcriptase (Invitrogen) according to manufacturer’s instructions. Total RNAs were extracted from labyrinthine areas (n = 5 in each group and sex) and livers (n = 5 in each group and sex) of different litters as described [28], then purified using RNeasy mini kit (Qiagen). Reverse transcription was performed for 1h at 37°C with 1 µg of RNA, 1.5 mM dNTPs, 150 ng Randoms hexamers (Invitrogen) and 200U M-MLV (Invitrogen) followed by one step at 70°C for 15min. The PCR reactions were carried out with SYBR green and 300 nM gene-specific primers (Table 1) on an ABI Prism TM 7000. To verify the amplicon specificity, amplicons were purified using a Wizard® SV gel and PCR clean-up system (Promega) and sequenced by Beckman and Coulter Genomics, then blasted against the rabbit genome. Data were analyzed using QbasePLUS software® (Biogazelle). To calculate the normalized relative quantity (NRQ), target and run specific amplification efficiencies were taken into account. *β-actin*, *EIF4A2* (Eukaryotic translation initiation factor 4E family member 2) and *RPL18* (Ribosomal protein L18) genes, as selected by geNorm, were used as reference genes for the placental and hepatic samples whereas *GAPDH* (Glyceraldehyde-3-phosphate dehydrogenase), *HRPT1* (Hypoxanthine phosphoribosyltransferase 1) and *PPIA* (Peptidyl prolyl cis/trans isomerase) were used for the blastocysts. Each NRQ value was divided by run and gene specific calibration factor to determine calibrated NRQ (CNRQ).

**Table 1:** Gene-specific primers and their accession number.

Gene	Forward and reverse primers	Accession number
*ABC-A1*	F 5'-GGTGATGAGCCGGTCAATG-3'	XM_002708133
	R 5'-CCATGATCCGCATGGTCTC-3'	
*ABC-G1*	F 5'-TCATCCTGTCCATCTACGGCC-3'	ENSOCUT00000027993
	R 5'-TGCAACTTGGCGTTCTCCAC-3'	
*ACC-α*	F 5’- CTAACAACGGCATTGCAGCA-3’	ENSOCUT00000021807
	R 5’- GGCTTTCAGGTCTTCAGGTGTG-3’	
*Adipophilin*	F 5'-GGGCCAGAGTTTCTGTAGCCA-3'	CU465044
	R 5'-CCCAAGACTGTGTTAATGCTGC-3'	
*βactin*	F 5'-CGAGACCACCTTCAACTCGATC-3'	NM_001101683
	R 5'-CTTCTGCATGCGGTCGG-3'	
*CD36*	F 5'-TGCTGCAGTTCTTTTCCTCTGA-3'	AF412572
	R 5'-GGAGATGCAAAAGCCTTGGC-3'	
*EIF4E2*	F 5'-TGGCAAGTGGATTATTCGGC-3'	ENSOCUG00000004534
	R 5'-CAGAGACCACAGCCCCACAG-3'	
*FAS*	F 5’- ACTACAACCTCTCGCAGGTGTG-3’	ENSOCUT00000022126
	R 5’- AGGGAGCTGTGCATGATGC-3’	
*FATP-4*	F 5'-AGGAGCTGCCCCTGTATGC-3'	XM_002722970
	R 5'-CACGACAGCTGGGTCAAAGC-3'	
*HMG-CoA reductase*	F 5'-GACTCCCCACACAGAGCTGC-3'	XM_002723033
	R 5'-ATTCTTCATTAGGCCGAGGCT-3'	
*LDL-R*	F 5'-GTGCAACTCCGCCAGGGA-3'	XM_002723277
	R 5'-GCCGATTCTGAGGTCGAAGC-3'	
*LXR-α*	F 5'-AGGATTTCAGCTACAACCGGG-3'	AB536719
	R 5'-GCGAACTCGGCATCATTGAG-3'	
*PPAR-γ*	F 5'-TGAACGACCAGGTGACTCTGC-3'	NM_001082148
	R 5'-TCCCTCGTCATGAAGCCTTG-3'	
*RPL18*	F 5'-CAACTCCACGTTCAACCAGGT-3'	DQ403030
	R 5'-GGTCTTGTTCTCCCGCCC-3'	
*RXR-α*	F 5'-CAAGGAGAGGAACGAGAACG-3'	AF136242
	R 5'-CACGTAGGTCTCGGTCTTGG-3'	
*SLC2A1*	F 5'-ACCACGCTGTGGTCCCTCT-3'	NM_001105687
	R 5'-GCAGGTTCATCATCAGCATTGA-3'	
*SLC2A3*	F 5'-AGAAGGAAGAGGACGAGGCC-3'	XM_002712761
	R- 5'-GTGACTTGCTTCTCCTGGGC-3'	
*SLC38A1*	F 5'-GCGTGCACACCAAGATACGT-3'	ENSOCUT00000005167
	R 5'-ACCGATCCTTAAGCTCGCTG-3'	
*SLC38A2*	F 5'-ATTGTCCGACTGGCTGTGCT-3'	ENSOCUT00000009942
	R 5'-CTGTGACGCCACCAACTGAA-3'	
*SLC38A4*	F 5'-GGATGCAGACGGTGTCCAAC-3'	ENSOCUT00000026469
	R 5'-CTGTAGGCGTGCAGCAGTTC-3'	
*SREBP2*	F 5'-GCTCGAGCCTCCCAAAGAAG-3'	AF278693
	R 5'-GGCATCTGTCCCCATGACC-3'	

### Immunofluorescence

On day 6, blastocysts (n = 41) were fixed in 4% of paraformaldehyde overnight at 4°C and permeabilized with 0.5% Triton X-100 for 30 min. Embryos were pre-incubated with 2% bovine serum albumin. Adipophilin antibody (Guinea pig polyclonal antibody, PROGEN®, 1∶100) was incubated overnight at 4°C and with Cy5 coupled secondary antibody (Jackson Immunoresearch, 1∶200) for 1 hour then rinsed and incubated with Nile Red 30 min (1∶500). DNA was counterstained with DAPI. After post-fixation in 2% of paraformaldehyde for 15min, embryos were mounted on slides and observed with a Zeiss ApoTome (MIMA2 platform).

### Cholesterol and triacylglycerol analysis in livers and labyrinthine areas

Livers (n = 5 in each group) and labyrinthine areas (n = 5 in each group and sex), randomly chosen from three litters and following a fine dissection of the placenta, were homogenized in methanol/5 mM EGTA with FAST-PREP (MP Biochemicals).

Complex lipids (cholesterol, cholesteryl esters and triacylglycerol) were extracted from 2 mg of placental tissue in dichloromethane/methanol/water in the presence of internal standards (3 mg of stigmasterol and cholesteryl heptadecanoate, 4 mg of glyceryl trinonadecanoate) [29]. The dichloromethane phase was evaporated to dryness and dissolved in ethyl acetate. One ml of the lipid extract was analyzed by gas-liquid chromatography on a FOCUS Thermo Electron system using a Zebron-1 Phenomenex fused silica capillary columns [30]. To express results in nmol/mg of proteins, proteins were measured. Five hundred µg of tissue was dehydrated by speed vacuum, dry pellets were dissolved in 0.1 M NaOH overnight and proteins were quantified with the Bio-Rad assay.

### Fatty acids (FA): Saturated fatty acids (SFA), Monounsaturated fatty acids (MUFA) and Polyunsaturated fatty acids (PUFA) analysis

One hundred µl of plasma (n = 6 in each group and sex), labyrinthine area and liver samples randomly chosen from each litters, were stored at –20°C in chloroform/methanol. Lipids corresponding to 400 mg of placental and hepatic tissue were extracted with CHCl_3_-MeOH [31]. The phospholipids were separated from the non-phosphorous lipids on silica acid cartridges. The phospholipid fraction, mostly representative of the membrane lipids, and the neutral lipid fraction, representative of the storage lipids were transmethylated with Boron trifluoride methanol 7% (Sigma-Aldrich) [32]. The methyl esters of phospholipid or neutral FA were analyzed by gas chromatography coupled to FID (Gas Chromatograph 3900 Varian) on an Econo-Cap EC-WAX capillary column using heptadecanoic acid (margaric acid, C17:0) as internal standard [33]. Results were expressed as g/l of plasma and mg/g of placenta or liver.

### Statistical analysis

To analyze the effect of diet on maternal metabolism, litter size, maternal total cholesterol, cholesteryl esters and triacylglycerol concentrations in livers, the non-parametric two-sample permutation test was performed using R statistical software and the package Coin including in R commander (R Core Team (2013) ; www.r- project.org/ ). Data are expressed as: median (Q1; Q3). The first quartile (Q1) and the third quartile (Q3) correspond respectively to twenty-five and seventy-five percent of scores.

For the analysis of biometric parameters (fetal weight and fetus to placenta-decidua weight ratio), a linear model was used, taking into account the maternal diet and the litter size as main variables, with random effect of the dam.

Sexual dimorphism, dietary effects or an interaction between these factors and fetal and placental data, hepatic function and gene expression were assessed by analysis of variance (ANOVA) using R software, after verification of the homogeneity of variance using the Levene test. Comparison between groups was performed using a Tukey’s *posthoc* test. P<0.05 was considered statistically significant and data are expressed as means ± standard error (SEM).

## Results

### The HH diet modified the maternal metabolism on D28 of gestation

On D28 of gestation, total maternal plasma cholesterol concentrations were significantly higher in HH does compared to C does (0.47(0.33;0.60) vs. 0.10 (0.09;0.17) g/l; p<0.001), whereas triacylglycerol concentrations were not statistically different between the two groups (1.35(0.85;2.62) vs. 0.87(0.36;1.07) g/l; p = 0.07). Maternal plasma fatty acids (FA) concentrations tended to be higher in HH compared to C does (1.53 (0.62;2.15) vs. 0.62(0.45;0.91) g/l; p = 0.051). The maternal plasma FA profile reflected the dietary FA intake with more n-6 PUFA in HH compared to C does (p<0.01)(Figure 1B).

### The HH diet induced intrauterine growth retardation in both sexes, which was associated with a dyslipidemia that affected more males than females at D28 of gestation

At D28 of gestation, litter size was not statistically different between HH and C does (8.0(7.0;10.0) and 8.5(6.7;9.0) fetuses, respectively), indicating that the HH diet did not affect implantation.

Altogether, 58 HH and 43 C fetuses were obtained. Sex ratios within the litters were not significantly different. Fetuses from HH does were significantly lighter (–17.8%) than C (29.1±0.9 vs. 35.4±1.2 g; p<0.001), whereas fetal to placenta-decidua weight ratio remained unchanged. No sexual dimorphism was observed for these measurements.

**Figure 2:**
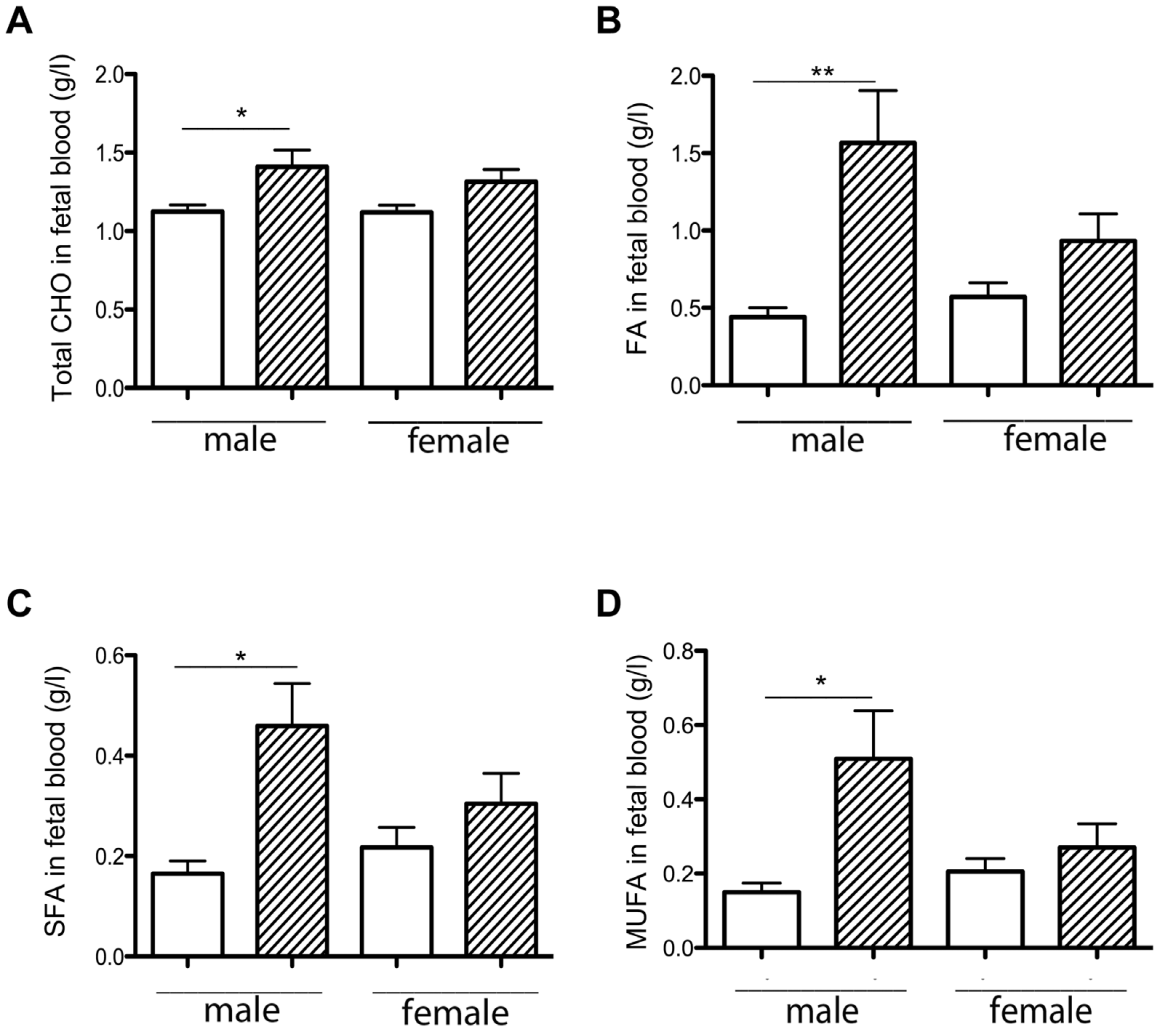
Effects of maternal diet on total cholesterol (A), FA (B), SFA (C) and MUFA (D) concentrations in fetal plasma on D28 of gestation, according to sex. For total cholesterol concentrations, 12 males and 11 female fetal samples from HH does (hatched columns) and 14 males and 12 females from C does (white columns) were used. For FA, SFA and MUFA concentrations, 6 samples by group were analyzed. (*p<0.05 and **p<0.01)

Fetal plasma samples were collected randomly from all does, resulting in 23 HH and 26 C fetuses. Total fetal cholesterol plasma concentrations were significantly increased (1.2 fold) in HH compared to C (1.36±0.07 vs. 1.12±0.03 g/l; p<0.01). This effect was due to an increase in HH males but not in females (Figure 2A). Fetal triacylglycerol plasma concentrations were significantly increased (2.3 fold) in HH compared to C fetuses (4.18±0.65 vs. 1.84±0.22 g/l; p<0.05), without sexual dimorphism. Total plasma FA concentrations were significantly increased in HH compared to C fetuses (1.25±0.2 vs. 0.51±0.06 g/l; p<0.001) and that was also the case for saturated fatty acids (SFA), monounsaturated fatty acids (MUFA) and PUFA (p<0.01, p<0.05 and p<0.001, respectively). Sexual dimorphism was observed for FA (Figure 2B), with significantly higher concentrations of SFA (Figure 2C) and MUFA (Figure 2D) in HH males compared to C males (p<0.01, p<0.05 and p<0.05, respectively), but not in females. For PUFA, plasma concentrations were increased in both HH males and females (p<0.001 and p<0.01 respectively).

The difference in lipid profiles between HH males and females could result from the modification of fetal hepatic metabolism and/or lipid transfer from the placenta to the fetal side, which were subsequently explored (see below).

### The maternal HH diet increased hepatic FA storage in female and male fetuses

To understand the fetal phenotype, the liver was studied at 28 days of gestation. Neutral lipid composition was determined in HH and C fetuses. No difference in cholesterol, cholesteryl ester and triacylglycerol concentrations was observed (data not shown). Hepatic FAs were analyzed, segregating between membrane phospholipids and intracellular lipid storage (cholesteryl esters and triacylglycerol).

**Figure 3:**
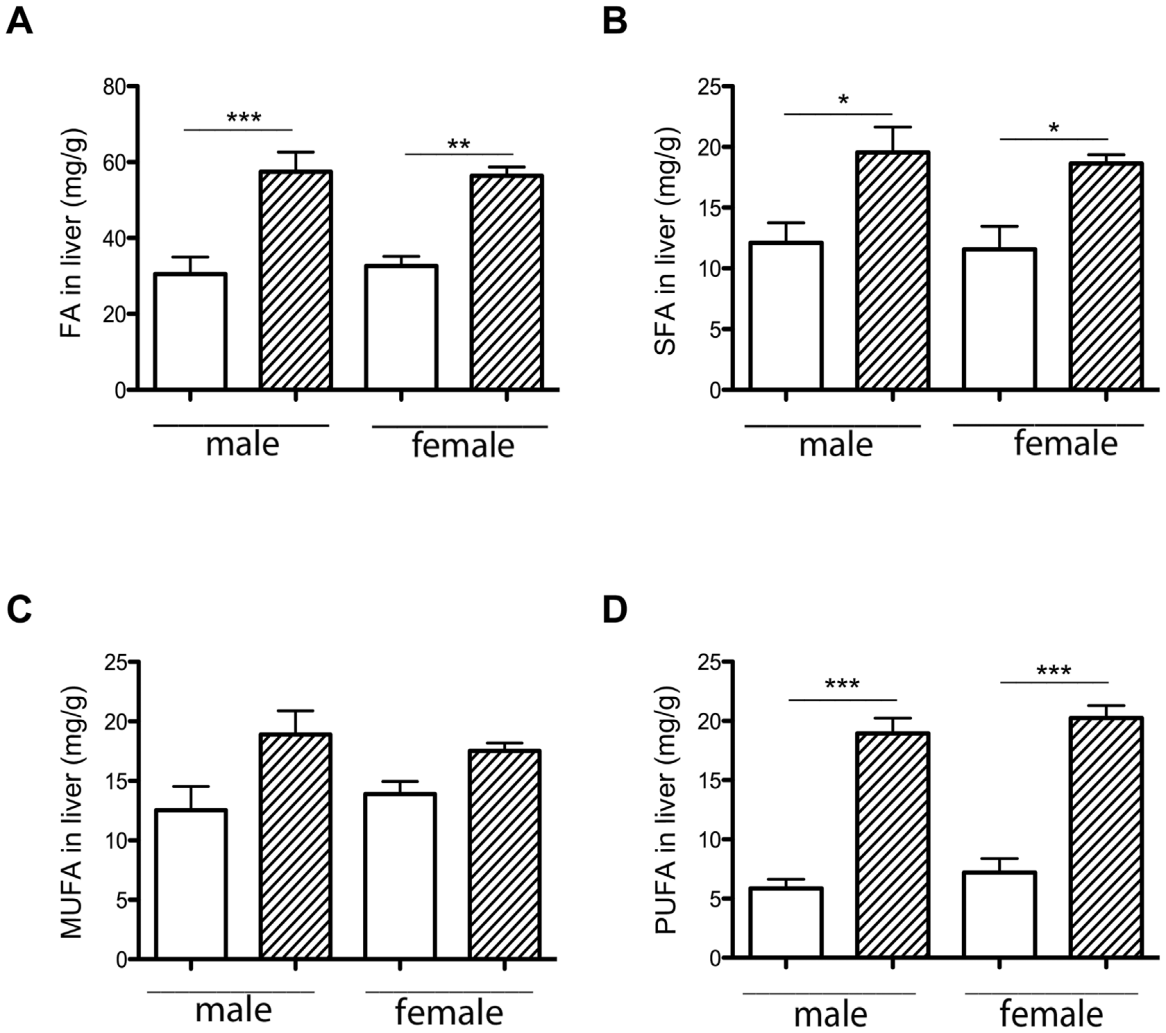
Effects of maternal diet on liver FA (A), SFA (B), MUFA (C) and PUFA (D) storage, according to sex. 6 samples per group were analyzed. HH is indicated by hatched columns and C diet by white columns. (*p<0.05, **p<0.01 and ***p<0.001).

In phospholipids, FA, SFA and MUFA concentrations were not significantly different in HH compared to C livers. There was a significant increase in PUFA concentrations in HH compared to C livers (3.76±0.19 vs. 2.88±0.17 mg/g of liver; p<0.01). No change in total FA content of the phospholipid fraction indicated that no hepatic hypertrophy occurred. Liver FA storage was significantly higher in HH compared to C livers (56.9±2.7 vs. 31.6±2.44 mg/g of liver; p<0.001) and in both HH males and females (Figure 3A). Both SFA and PUFA concentrations were significantly increased in HH compared to C livers (1.6 fold, p<0.05; 3 fold, p<0.001, respectively), but not MUFA. The SFA and PUFA concentrations were significantly higher in the liver of both HH males and females compared to their respective controls (Figure 3B and 3D), but MUFA remained unchanged (Figure 3C).

The expression of genes involved in lipid and cholesterol metabolism was analyzed (Table 2). Relative gene expression of *ACC-α* (Acetyl-CoA carboxylase), which is a limiting enzyme in de novo fatty acids synthesis, was decreased (–17.04%) in HH livers compared to C livers (p<0.01). The transcripts of *FAS* (Fatty Acid Synthase) involved in fatty acid synthesis, and that of *FATP-4* (Fatty Acid Transport Protein), *Adipophilin, PPAR-α* (Peroxisome Proliferator-Activated Receptor), *LXR-α* (Liver X receptor), *SREBP-2* (Sterol Regulatory Sterol Regulatory Element-Binding Protein), *HMG-coA reductase* (Hydroxymethylglutaryl-Coenzyme A reductase), which are involved in several aspects of lipid metabolism, remained unchanged (Table 2). In contrast, the expression of the *LDL-R* (LDL-Receptor) was decreased (–16.76%) in HH compared to C livers (0.91±0.03 vs 1.1±0.06 A.U.; p<p0.05). No sexual dimorphism was observed.

**Table 2:** Relative gene expression in livers according to the maternal diet.

Gene	C liver	HH liver	P value
*ACC- α*	1.1±0.06	0.91±0.03	p<0.01
*Adipophilin*	1.66±0.43	0.85±0.13	NS
*FAS*	1.00±0.15	1.00±0.09	NS
*FATP-4*	1.00±0.07	1.07±0.13	NS
*HMG-CoA reductase*	1.01±0.03	0.97±0.05	NS
*LDL-R*	1.01±0.07	0.91±0.03	p<0.05
*LXR-α*	1.06±0.08	0.91±0.05	NS
*PPAR- α*	1.08±0.07	0.92±0.07	NS
*SREBP-2*	0.95±0.07	0.95±0.04	NS

Ten livers per group were used. Values are expressed as means ± standard error (SEM).

### The HH diet induced changes in the trophoblastic layer of the blastocyst and the placental phenotype, with sexual dimorphism

**Figure 4:**
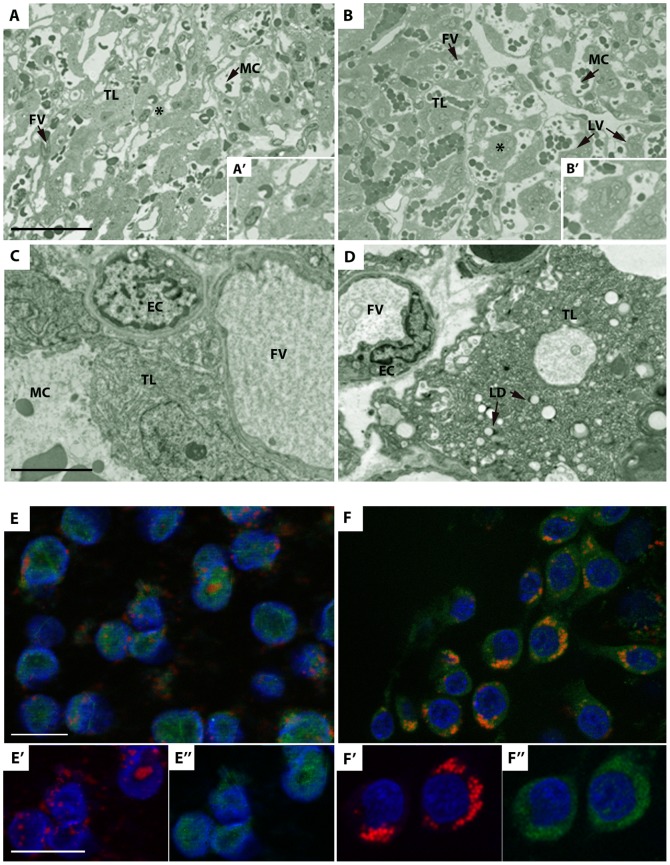
Morphological analysis of placentas on D28 of gestation and distribution of lipid droplets on D6 in blastocysts. (A-B) Light microscopy morphological analysis of placentas on D28 from C and HH fetuses. Numerous light vesicles (LV) are located in the trophoblastic layer (TL) of HH (B) compared to C placenta (A). A’ and B’ represent the high magnification of the zone indicated by a star. Scale bar: 100 µm. (C-D) Transmission electron microscopy of placentas on D28 of gestation from C and HH fetuses. Light vesicles were identified as lipid droplets (LD) in the trophoblastic layer (TL) of HH (right panel) compared to C placenta (left panel). EC: Endothelial cell; FV: Fetal Vessel; MC: Maternal Compartment. Scale bar: 5 µm. (E-F) Distribution of lipid droplets on D6 in C and HH blastocysts Fluorescent immunodetection of adipophilin (green), Nile red (red), and DNA (blue) from C (E) and HH (F) blastocysts. In HH blastocysts, abnormal accumulation of lipid droplets is observed around the nuclei (F’), colocalized with adipophilin staining (F’’), in contrast to C blastocysts (E-E”). Scale bar: 20 µm.

As neither the fetal hepatic function nor its lipid content could account for the blood profiles in HH fetuses, the placenta, which is essential for nutrient supply, was studied. At D28 of gestation, placental morphology (labyrinthine area) was analyzed by light and transmission electron microscopy (Figure 4A-D). In the labyrinthic zone (which is involved in placental exchange), numerous light vesicles were observed in the cytoplasm of the trophoblastic layer in the HH placentas (Figure 4B-B’) in contrast to the C placentas (Figure 4A-A’). Ultrastructural analysis (Figure 4C-D) indicated that these light vesicles were accumulated lipid droplets (Figure 4D). At the blastocyst stage, using immunodetection of adipophilin in association with Nile-Red to stain lipids, an accumulation of lipid droplets was also observed in the cytoplasm of the trophoblastic cells in HH blastocysts (Figure 4F-F’’), but not in C (Figure 4E-E’’).

**Figure 5:**
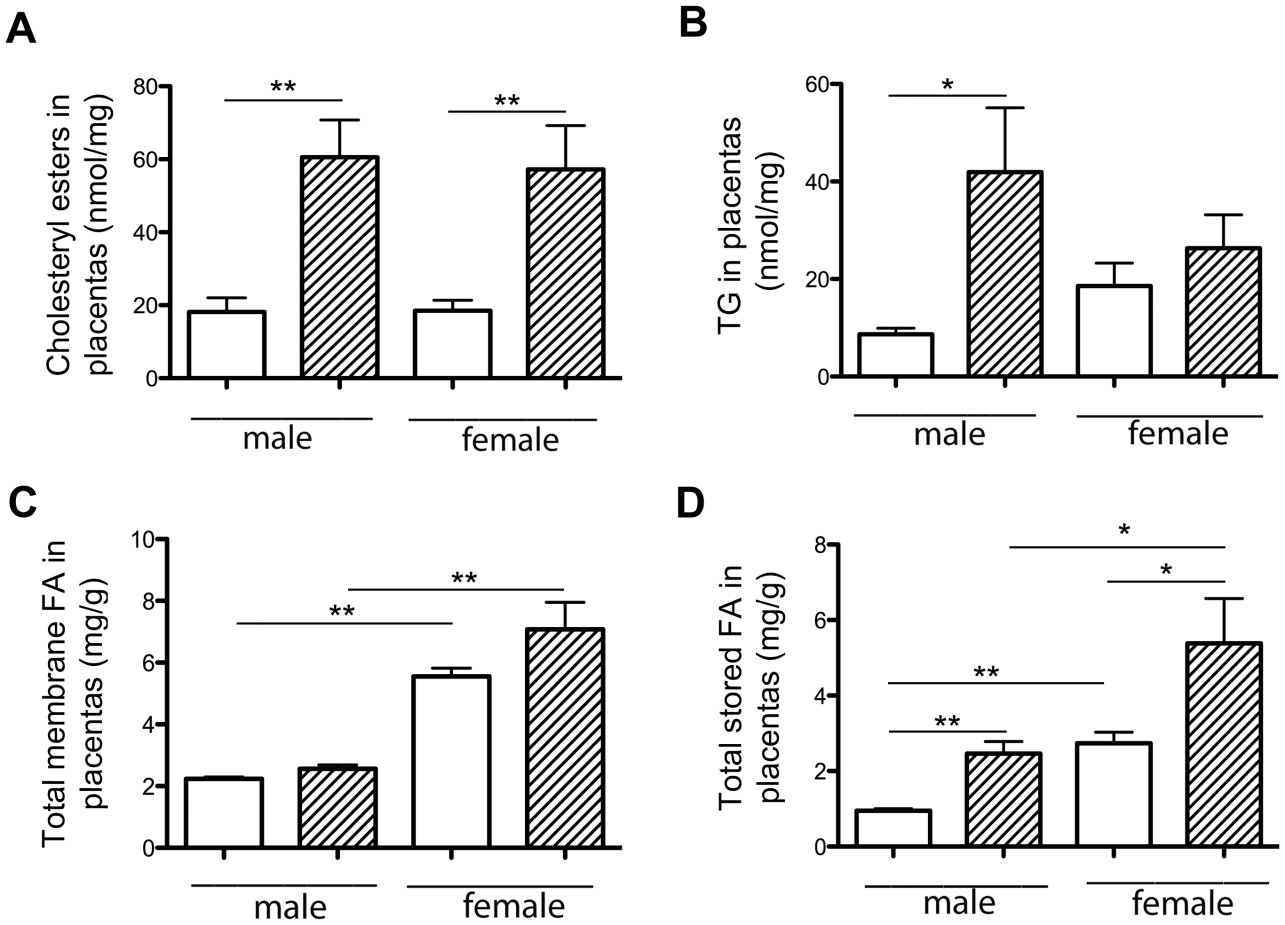
Cholesteryl esters (A), triacylglycerol (B), membrane (C) and storage lipids (D) concentrations in C and HH placentas on D28 of gestation, according to fetal sex. For cholesteryl esters and triacylglycerol (TG) concentrations, 5 samples were used per group, whereas for FA concentrations, 6 samples per group were analyzed. HH is indicated by hatched columns and C diet by white columns. (*p<0.05 and **p<0.01).

Total placental cholesterol concentration remained unchanged between HH and C groups (51.04±2.9 vs. 44.9±1.7 nmol/mg of proteins). In contrast, the concentration of total cholesteryl esters was significantly increased by 3.2-fold in HH compared to C placentas (58.9±7.44 vs. 18.3±2.28 nmol/mg of proteins; p<0.001) in both males and females (Figure 5A) with a HH/C ratio equal to 3.33 and 2.79, respectively. Triacylglycerol concentrations were significantly increased in HH compared to C placentas (34.1±7.49 vs. 13.6±2.83 nmol/mg of proteins; p<0.01), with an increase in HH vs. C males but not females (Figure 5B). Consequently, the HH/C ratio was much higher in males than in females (4.8 and 1.4, respectively).

**Figure 6:**
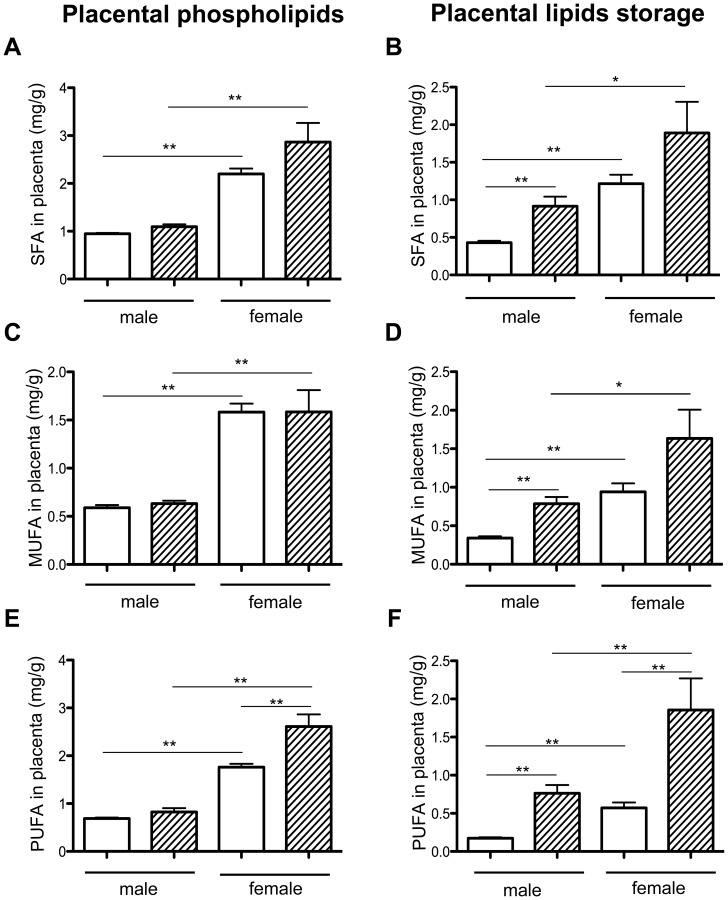
Placental quantification of SFA, MUFA and PUFA from membrane phospholipids and intracellular lipid storage expressed in mg/g of placenta in HH and C according to fetal sex. Six placentas per group were analyzed. Values are expressed as means ± standard error (SEM). (*p<0.05 and **p<0.01).

Placental FA were analyzed, segregating between placental phospholipids (placental membrane) and neutral lipids (lipid storage). Placental phospholipid contents were not different between HH and C groups. There was a significant increase in total FA contents of placental membranes in C females compared to C males (2.47 fold), indicating sexual dimorphism, but also in HH females compared to HH males (2.76 fold) (Figure 5C). The HH/C ratio of male and female lipids were close (1.1 and 1.2, respectively), indicating that the HH diet does not affect placental membranes. SFA, MUFA and PUFA concentrations were significantly increased in C females compared to C males (Figure 6A, 6C and 6E) but also in HH females compared to HH males. Moreover, there is a significant difference in PUFA concentrations between HH and C females (p<0.01). The HH/C ratio of SFA males and females were almost similar (1.2 and 1.3) as well as the HH/C ratio of MUFA (1.07 in males and 1 in females) and PUFA (1.2 in males and 1.5 in females, respectively). These data suggested that maternal HH diet affected placenta membrane PUFA of females only.

In terms of placental FA storage, there were significantly more FA in HH compared to C placentas (3.9±0.7 vs. 1.84±0.3 mg/g of placenta; p<0.01), with higher concentrations in females compared to males in both in C and HH groups (Figure 5D), indicating a sexual dimorphism. The HH/C ratio in males and females was 2.6 and 1.96, respectively. These data suggest an effect of maternal HH diet on FA storage. SFA, MUFA and PUFA concentrations were significantly increased in HH compared to C placentas (SFA and MUFA, p<0.05; PUFA, p<0.001). SFA, MUFA and PUFA were significantly different in HH males compared to C males, but also in C females compared to C males and in HH females compared to HH males (Figure 6B, 6D and 6F), indicating a sexual dimorphism. Moreover, PUFA increased significantly in HH females compared to C females and in HH males compared to C males (Figure 6F). The HH/C ratio was determined for SFA, MUFA and PUFA. The SFA ratio was equal to 2.12 in males and 1.55 in females, those of MUFA were 2.52 and 1.73, respectively. PUFA ratio was much higher in males (4.36) than in females (3.23). These data indicated that the maternal HH diet impacted concentrations of SFA and MUFA in males and PUFA concentrations in both sexes. SFA, MUFA and PUFA concentrations, however, were always much higher in HH females compared to HH males.

### The HH diet modified the placental gene expression in the labyrinthine area in a sexually dimorphic manner

**Figure 7:**
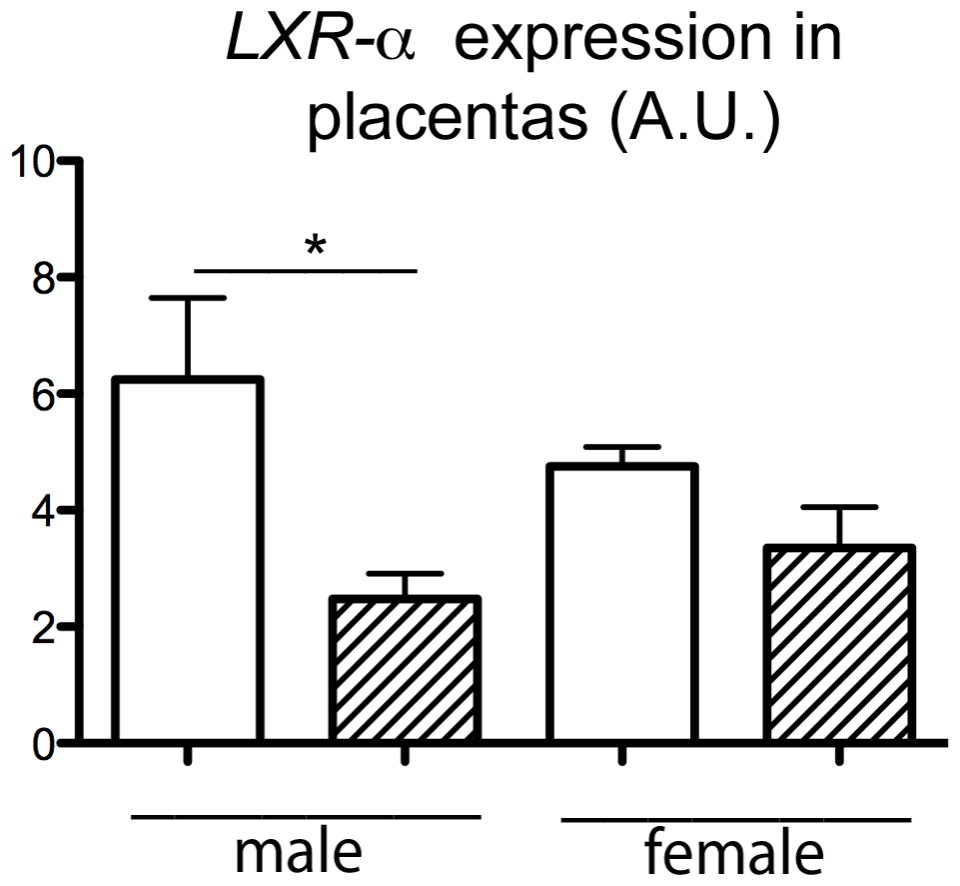
Relative expression of *LXR-α* expressed in arbitrary unit (A.U.) in HH and C placentas, according to sex. N = 5 per group. HH is indicated in hatched columns and C diet by white columns.

The IUGR and the dyslipidemia observed in HH fetuses, together with a high placental accumulation of lipids strongly suggested that the expression of placental genes involved in nutrient transfer and metabolism could be altered. Consequently, the expression of a selected number of relevant genes involved in transplacental transfers was quantified using RT-qPCR (Table 3). Transcripts of glucose transporters including *SLC2A1* (Solute carrier family 2) and *SLC2A3* were not modified by maternal diet. In contrast, *SLC38A1* (Solute carrier family 38), which is involved in neutral amino acid transport, was significantly downregulated in HH compared to C placentas (p<0.05) whereas the other transporters *SLC38A2* and *SLC38A4* transcripts were not affected by maternal diet. For lipid metabolism and transport, the expression of *LDL-R*, *CD36* (Fatty acid translocase) and *LXR-α* genes, which are involved in the cholesterol exchange pathways, was significantly decreased in HH compared to C placentas (p<0.01). Sexual dimorphism was observed only for *LXR-α* mRNA that was significantly downregulated in the placenta of HH males but not females (p<0.05) (Figure 7). The cholesterol transporter *ABC-G1* (ATP binding cassette transporter), controlled notably by *LXR-α,* was also down regulated in the HH group (p<0.05) but *ABC-A1, HMG-CoA reductase* and *SREBP-2*, which are also involved in the cholesterol trafficking pathways remained unchanged. Concerning lipid metabolism, the expression of *adipophilin*, *FATP-4*, *PPAR-γ* and its partner *RXR-α* (Retinoid X Receptor) was investigated but there was no difference between groups.

**Table 3:** Relative gene expression in placentas according to the maternal diet.

Gene	C placenta	HH placenta	P value
*ABC-A1*	1.09±0.09	1.04±0.09	NS
*ABC-G1*	1.29±0.11	0.84±0.13	p<0.05
*Adipophilin*	1.03±0.10	1.38±0.27	NS
*CD36*	1.36±0.08	0.98±0.08	p<0.01
*FATP-4*	1.3±0.17	1.08±017	NS
*HMG-CoA reductase*	1.12±0.10	0.98±0.07	NS
*LDL-R*	2.01±0.26	0.86±0.19	p<0.01
*LXR-α*	5.5±0.72	2.92±0.41	p<0.01
*PPAR-γ*	1.43±0.22	1.25±0.14	NS
*RXR-α*	0.93±0.15	1.37±0.19	NS
*SLC2A1*	1.09±0.13	0.92±0.04	NS
*SLC2A3*	0.87±0.07	0.98±0.12	NS
*SLC38A1*	1.04±0.06	0.82±0.06	p<0.05
*SLC38A2*	0.95±0.07	0.78±0.07	NS
*SLC38A4*	0.95±0.17	1.01±0.15	NS
*SREBP2*	0.84±0.10	0.78±0.12	NS

Ten placentas per group were used. Values are expressed as means ± standard error (SEM).

### HH diet affected the gene expression at the blastocyst stage

Several genes analyzed in placentas were investigated at the blastocyst stage (Table 4). Relative expression of *SLC2A1* and *SLC2A3* increased significantly in all HH compared to all C blastocysts (p<0.05 and p<0.001, respectively). Transcripts of *SLC38A2* were significantly decreased in HH compared to C blastocysts (p<0.05) whereas *SCL38A1* remained unchanged. Transcripts of *LXR-α* and *Adipophilin* were significantly reduced in HH compared to C blastocysts (p<0.05 and p<0.01, respectively). Relative expression of *ABC-G1* was significantly increased in HH compared to C blastocysts (p<0.001), whereas *ABC-A1, CD-36, LDL-R* and *PPARγ* remained unchanged.

**Table 4:** Relative gene expression in blastocysts according to the maternal diet.

Gene	C blastocyst	HH blastocyst	P value
*ABC-A1*	1.09±0.09	1.02±0.10	NS
*ABC-G1*	0.86±0.04	1.18±0.08	p<0.001
*Adipophilin*	1.08±0.04	0.95±0.03	p<0.01
*CD-36*	0.95±0.04	1.06±0.05	NS
*LDL-R*	0.96±0.04	1.06±0.06	NS
*LXR-α*	1.2±0.13	0.91±0.05	p<0.05
*PPAR-γ*	1.04±0.12	1.11±0.15	NS
*SLC2A1*	0.94±0.03	1.07±0.04	p<0.05
*SLC2A3*	0.87±0.02	1.15±0.05	p<0.001
*SLC38A1*	1.09±0.05	0.96±0.06	NS
*SLC38A2*	1.07±0.03	0.95±0.03	p<0.05

Eleven blastocysts from C dams and thirteen blastocysts from HH dams were analyzed. Values are expressed as means ± standard error (SEM).

## Discussion

Excessive fat intake has become a major health concern in western countries, with women of childbearing age being largely exposed to high fat diets. Here, using a previously developed rabbit model, we observed that a maternal HH diet affected the trophoblast at the blastocyst stage and also gene expression in the blastocyst. Later in pregnancy, all HH fetuses were growth retarded. Specific sexually dimorphic changes were observed in the placenta not only in terms of gene expression but also in terms of physiological adaptation, with a relative protection of the female fetuses from developing dyslipidemia, as demonstrated by an increased lipid storage in female placentas compared to males, while the HH males exhibited higher plasma FA and cholesterol concentrations compared to females.

On day 28, the maternal HH diet induced high maternal plasma PUFA and cholesterol concentrations. These data indicate that maternal plasma composition is related to maternal dietary fatty acids composition, as described by Amusquivar [34]. Moreover, the fold-change of total cholesterol concentrations observed between HH and C does in the present study is in the same range as those reported in a previously published rabbit model fed with a cholesterol-enriched diet supplemented or not with lipids [8], [9], [16]. The present data are also in agreement with that reported at 30 days of gestation in females fed a diet supplemented with 0.2% cholesterol [16].

This adverse maternal environment induced an abnormal accumulation of lipid droplets in the trophoblastic layer of HH placentas, which is involved in materno-fetal exchanges, consistent with macroscopic observations described by others using a 0.2% cholesterol diet [16]. Lipid droplets are dynamic organelles known to store neutral lipids (triacylglycerol and cholesteryl esters). Here, lipid droplet accumulation correlated with the presence of large amounts of cholesteryl esters, triacylglycerol and stored FA in HH placentas without any change in total cholesterol contents. This could be linked to an increase in the uptake of maternal lipoproteins through the placenta as described in female hamsters [35], since maternal plasma HDL concentrations are increased at mid-gestation in HH does in the present model [9]. Maternal lipoproteins, including cholesterol, are up-taken by the trophoblast. Cholesterol effluxes from the placenta to the fetal side via adenosine triphosphate-binding cassette transporters (ABC), located on the basal membrane of the trophoblast [36]. As over accumulation of free cholesterol can be toxic to cells, cholesterol is converted to cholesteryl ester through acyl-coenzyme A: cholesterol acyltransferase (ACAT), as reflected by increased concentrations of cholesteryl ester in both male and female HH placentas. Cholesterol concentrations, however, only increased in the plasma of HH males. Consequently, the observed sexual dimorphism could be attributed to a difference in ACAT activity in male and female HH placentas, even though *ACAT* gene expression was unchanged (data not shown). The sequestration of FA by female HH placentas suggests a relative protection of females against dyslipidemia and lipotoxicity compared to males. Interestingly, the increase in SFA and MUFA storage in HH female placentas in response to the HH diet was much higher than in males. Consequently, the placenta seemed to act as a protective barrier in females but did not entirely succeed in this role since the females exhibited fatty liver as well as males. Moreover, the placenta is known to preferentially transfer PUFA, due to their absolute requirement for brain and retina development [37]. This feature could explain the high levels of PUFA in the plasma of HH fetuses, the increased PUFA placental storage in both sexes and PUFA accumulation in the liver, both in membranes and intracellular lipid storage. This accumulation could explain the decrease in *ACC-α,* which is a limiting enzyme in *de novo* fatty acids synthesis. These data are consistent with the regulation of this enzyme described by Mao et al. [38].

The expression of genes involved in FA and cholesterol transport and metabolism was investigated in the blastocyst stage and in the placenta. Previous studies in our laboratory demonstrated a higher expression of *adipophilin*, a lipid droplet coat protein, at the 8-16 cell stage embryo [9]. Surprisingly, on day 6, *adipophilin* was downregulated in HH blastocysts, although numerous lipid droplets were observed in their trophoblastic layer. As the decrease of adipophilin is known to reduce lipid droplet formation [39], it is possible that a regulation loop occurred rapidly between the 8–16 cell and the blastocyst stage in order to limit lipid droplet accumulation. The regulation of adipophilin could be an adaptive response to the maternal HH environment, possibly reflected by oviductal fluid contents, although little data are so far available on the effects of a hyperlipidic environment on oviductal fluid composition [40]. Gene expression and lipid contents of embryos are known to be affected by maternal hyperlipidemia [12], [41], but the observed changes could also be due to changes in oocyte quality in response to altered follicular fluid contents [42], [43] or to altered oocyte maturation, which was shown to lead to adverse postnatal outcomes, although sex related effects were not investigated [11], [44]. At D28, accumulation of lipid droplets was reported in trophoblastic cells but without any modulation of mRNA for *adipophilin* in the labyrinthine area, whereas the accumulation of lipid droplets in the trophoblast of HH blastocysts compared to C controls was associated with a decrease of *adipophilin* mRNA in the total blastocyst. Most published data related to placental fatty acid transfer has been obtained from *in vitro* experiments using primary trophoblastic cells or placental cell lines. Using the BeWo cells (trophoblastic cell line) incubated with long-chain PUFA, Tobin et al., 2006 [45] have established that a time-dependent increase in *adipophilin* mRNA expression occurred with a maximal level expression after 9h followed by a slow decrease of expression after 24 and 48h of treatment. *In vivo*, a transitory regulation loop of *adipophilin* transcript could occur in the placenta during gestation as trophoblastic layer is in contact with maternal blood enriched in PUFA, followed by a return to its baseline near term. Moreover, as maternal diet was shown to affect the types and quantities of lipid droplets associated to protein composition in the liver [46], a switch of adipophilin toward another coating protein at the lipid droplet surface could occur in the placenta and explain the absence of deregulation in adipophilin expression at D28.

*LDL-R* transcripts were downregulated only in HH placentas, suggesting that LDL internalization was limited to reduce cholesterol and cholesteryl esters uptake, consistent with observations made in the human placenta [47]. The transcription factor LXR is responsible for protecting cells from cholesterol overload. Here, we found a downregulation of *LXR-α* expression at D6 in HH embryos, indicating a disturbance in cholesterol homeostasis during preimplantation development. Interestingly, similar observations were made in placentas collected from women suffering of preeclampsia, a human pregnancy disorder associated with hyperlipidemia and hypertension [48]. Since *LXR-α* was significantly downregulated in male HH placentas, this could be a male-specific adaptive response to cope with the high cholesterol concentrations observed in the plasma of HH males and to limit cholesterol efflux from the placenta to the fetal side. As LXRs regulate the transcription of genes involved in lipid metabolism such as *ABC-A1*, *ABC-G1* and a fatty acid translocase (*CD36*) [48], [49], we further focused on the expression of these genes. Transporters ABC-A1 and ABC-G1 are able to induce cholesterol efflux from cells. In human placenta, ABC-A1 protein expression is apical and involved in cholesterol efflux from the placenta to the maternal side, whereas ABC-G1 is expressed basolaterally and plays a role in cholesterol efflux to the fetus [50]. On day 6, *ABC-G1* was increased in HH blastocysts, therefore preventing the accumulation of cholesterol and/or oxysterols, which can be cytotoxic, especially at high concentrations [50]. In HH placentas, the decrease in *ABC-G1* mRNA could limit the transfer of cholesterol and its sterol metabolites to the fetal side, which is in agreement with the storage of cholesteryl esters observed in HH placentas. Downregulation could be attributed to the observed decrease in *LXR* expression [51]. Finally, CD36 is located on both apical and basal membranes of the placenta and interacts with several ligands (FA, oxidized LDL, collagen) [37]. In the liver, CD36 is a direct target of PPAR but also of LXR [52]. The decrease in *CD36* expression in HH placentas probably results from the decrease of *LXR-α* and limits FA and/or oxidized LDL uptake by HH placentas. The absence of sexual dimorphism in *ABC-G1* and *CD36* expression in HH placentas does not exclude the possibility of a difference in activity between both sexes. Altogether, these data suggest that the placenta modulates cholesterol trafficking/metabolism, in a sexually dimorphic manner, with a male specific adaptive response. Our data are consistent with those obtained in placenta from mice fed with maternal low or high fat diets which induce sex-specific dimorphic gene expression signatures with different biological functions involved [17], [18].

The placental studies provide several explanations about the IUGR in HH fetuses. First, maternal diet altered placental membrane PUFA composition in HH females, which is likely to induce changes in membrane fluidity [53] affecting membrane protein function. Thus ion channels, transporters and receptors function could be disturbed and impair nutrient transfer. Second, glucose transporters were only affected by the HH diet at the early blastocyst stage. In the mouse blastocyst, SLC2A1 is localized at the basal and basolateral trophoblast membrane to supply glucose to the ICM, whereas SLC2A3 is expressed in the outer apical membrane of the trophoblast to mediate glucose transport from the uterine fluid into the blastocyst [54]. Here, the increase in *SLC2A* could stimulate glucose uptake in HH embryos, leading to a delay in development [55]. This hypothesis is supported by our previous studies, where IUGR was observed by ultrasound as early as day 9 of pregnancy [9]. Conversely, *SLC38A* are downregulated in both HH blastocysts and placentas. This downregulation could contribute to the IUGR phenotype, since a decrease in system A activity, involved in amino acid transfer, in placenta in humans and animal models has been associated with IUGR [56], [57].

In conclusion, this study demonstrates that trophoblastic adaptive strategies in response to maternal hyperlipidemia start at a very early stage of development and that these adaptive mechanisms persist throughout gestation. In the present study, female placentas appeared to favor FA accumulation in the trophoblast, to protect their female fetuses from a rise in plasma lipids. In males, a reduction of cholesterol efflux from the placenta is favored at the transcriptomic level but nevertheless, the male fetuses displayed dyslipidemia. More detailed knowledge on these sexually dimorphic adaptive mechanisms in response to a maternal high fat diet will help us explain different sex-specific pathologies at adulthood. Moreover, precise phenotypic characterizations from early to late stages in conceptus development may lead to the proposal of better nutritional recommendations to childbearing age or pregnant women.
